# Training technical or non-technical skills: an arbitrary distinction? A scoping review

**DOI:** 10.1186/s12909-024-06419-6

**Published:** 2024-12-18

**Authors:** Maria Louise Gamborg, Lisa Beicker Salling, Jan Duedal Rölfing, Rune Dall Jensen

**Affiliations:** 1 MidtSim, Central Denmark Region, Hedeager 5, Aarhus, 8200 Denmark; 2 Department of Clinical Medicine, Faculty of Health, Aarhus University, Hedeager 5, Aarhus, 8200 Denmark; 3 Department of Orthopaedics, Aarhus University Hospital, Palle Juul-Jensens Boulevard 99, J801, Aarhus, 8200 Denmark

## Abstract

**Introduction:**

Medical education often aims to improve either technical skills (TS) or ‘non-technical skills’ (NTS) and how these skills influence adverse events and patient safety. The two skill sets are often investigated independently, and little is known about how TS and NTS influence each other. In this scoping review, we therefore aim to investigate the association between TS and NTS.

**Method:**

We conducted a scoping review of four databases in order to summarize, analyse, and collate findings from the included studies.

**Results:**

In total, 203 of 2676 identified studies were included in the final analysis. The first study was published in 1991, but the majority of studies were published in the last decade. The majority were intervention studies including 41 randomized controlled trials. The the objective structured assessment of technical skills (OSATS) was the most common assessment tool with strong validity evidence within TS, but many variations without validity evidence were used. Conversely, Non-Technical Skills for Surgeons (NOTSS) was the most used tool with strong validity evidence for assessing NTS. However, the majority of studies used non-validated self-assessment tools to investigate NTS. The correlation between TS and NTS was assessed in 46 of 203 studies, whereof 40 found a positive correlation.

**Discussion:**

Our findings echo previous literature suggesting that empirical literature investigating the interaction between TS and NTS lack methodological depth. In this review only a minority of the identified studies (*n* = 46) investigated this correlation. However, the results strongly indicate a correlation between TS and NTS skills, suggesting that physicians who are proficient in their NTS, also perform well on their TS. Thus, the distinction between them in learning designs may seem arbitrary. While this result is promising, the limited methodological rigour indicates a lack of proper understanding of NTS and how to properly assess them.

**Supplementary Information:**

The online version contains supplementary material available at 10.1186/s12909-024-06419-6.

## Introduction

A competent physician needs a broad variety of skills. While the needed competences are multifaceted, skill-based training in medicine often has a narrow focus. Skills are typically divided into technical skills (TS), such as the body-kinetic performance, and non-technical skills (NTS), often referring to human factors such as communication, decision making, leadership, and teamwork [1, 2]. In recent years, human factors gained notoriety for its negative impact on medical performance and threat to patient safety in terms of how these non-technical skills are impacted by environmental factors [3–8]. Thus, research on training initiatives targeting NTS are rapidly emerging in medical education. Here, simulation-based training has been investigated in relation to promoting clinical confidence [9], knowledge [10], collaboration [11], or overall NTS [12]. Similarly, other methods such as team interventions’ effect on team performance [13], and the effect of coaching on well-being and NTS [14] have also been investigated. This rising acknowledgement of NTS has been recognized by stakeholders. In example, employers of veterinarians report that NTS are an equally important feature in their employees, as technical skills [15].

In educational research outside the medical sphere, an interdependence of TS and NTS is well-established [16, 17]. While several authors have pointed towards this interdependence in medical education [18–21], it is less common to investigate the interaction between them. Research often assesses one of the two skills, but studies have found that both skill sets influence each other either directly or indirectly [22, 23].

Understanding the association between TS and NTS may help lay the foundation for more robust education of healthcare professionals, thereby improving overall treatment and enhancing patient safety. To date, only a couple of reviews investigating both TS and NTS have been performed in medical education, but often, they single out a specific phenomenon or medical specialty. Examples of these are reviews of training tools in ophthalmology [24] and urology [25], on the connection between spatial abilities and TS [26], the role of simulation-based training to promote TS and NTS [27, 28], and NTS in surgery and surgical teams [29–32]. Thus, none has, to our knowledge, performed a comprehensive review on the association between NTS and TS in medical education, overall.

The aim of this scoping review was therefore to explore the association between TS and NTS in medical education.

## Materials and methods

A scoping review was conducted following the methodological steps outlined by Arksey and O’Malley [33] and PRISMA for Scoping Reviews [34].

### Identifying relevant studies

Searches were made on four electronic databases: Embase, PubMed, Web of Science, and Scopus. The search string consisted of a variety of words related to “assessment”, “technical or non-technical skills”, “learning or education or training”, and “medicine or surgery or medical” in abstract or title. This was done to ensure that all studies were related to medical education and had an element of skill assessment. No start date was specified, as the inclusion of studies published over a relevant time span was considered essential for our study. The most recent search was conducted on December 4, 2023, in PubMed.

## Study selection

All identified studies were uploaded to EndNote and duplicates were removed. These were then transferred to Rayyan.ai [35] for sorting. Inclusion criteria were:

- Peer reviewed studies conducted in the Health Profession Education
- Inclusion of both technical and non-technical skills
- Assessment of skills

Exclusion criteria were defined during the analysis of the studies based on increased knowledge on the subject.

Overall, exclusion criteria were:

1. only assessment of either technical or non-technical skills
2. book chapters or short commentaries
3. non-English studies
4. non-health care studies
5. non-published studies
6. study protocols
7. reviews and conference abstracts

One author (LBS) reviewed all studies and removed duplicates. Three blinded reviewers (RDJ, MLG, LBS) independently reviewed the abstracts of a subset of 100 studies. All authors met to discuss inclusion and exclusion of publications, until consensus was reached. After consensus, the remaining studies were full-text screened by one author (LBS) and were either excluded or included in the final analysis.

### Charting the data

Key information for each included study was extracted using a charting form. This included: Author(s), year of publication, method(s), setting, participants/population, aim of the study, which NTS and TS was being assessed, what assessment tool was used to assess NTS and TS, main findings, conclusion, correlation between NTS and TS, and which medical specialty was represented in the study. In the present review, self-assessment is used as an umbrella term to cover different self-reported NTS.

Publications were categorized into 3 study types:

- intervention studies which describe and assess an intervention,
- explorative studies seeking to validate or explore coherence of NTS and TS,
- observational studies assessing performance by observing in-clinic behaviour.

## Collating, summarizing, and reporting the results

We collated and summarized the data in different illustrations depicting the yearly distribution of published literature, methods and study designs, assessment tools used in the assessment of NTS and TS, and finally quantity of studies finding a correlation between NTS and TS.

## Results

**Fig. 1:**
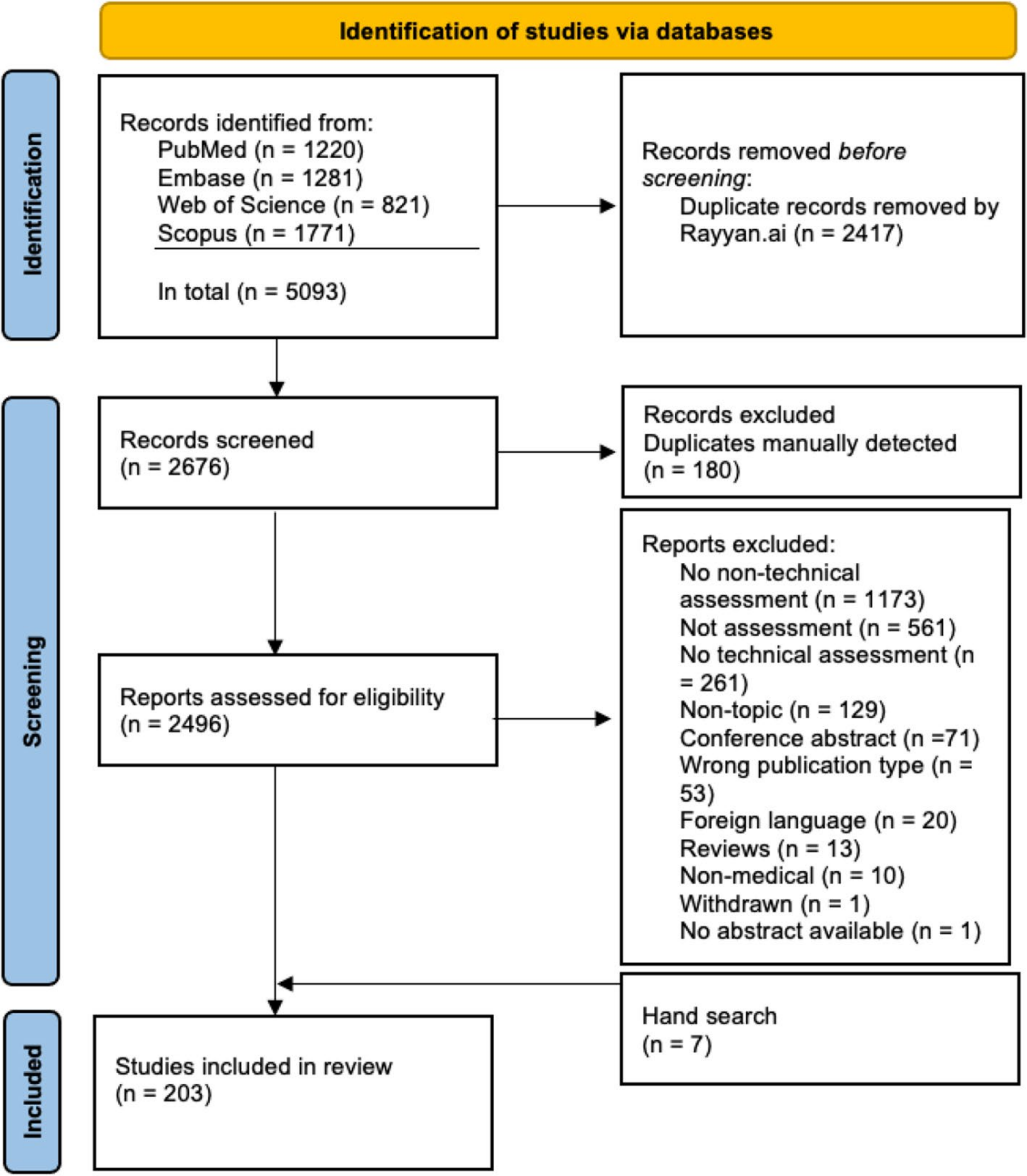
Flow chart for studies included and excluded in the study

We identified 5093 studies matching our criteria. After removal of duplicates in Endnote, 2676 studies were uploaded to Rayyan.ai [35], and another 180 manually identified duplicates were removed. The abstracts of the remaining 2496 studies were screened for eligibility. A total of 2218 studies were excluded as shown in the PRISMA flowchart (Fig. 1). The remaining 278 publications were full text screened, whereof 71 conference abstracts and 11 reviews were detected and excluded due to the lack of original empirical data. Seven additional publications were included after screening reference lists of included studies.

In total, 203 publications were included in the final analysis. An overview of these publications is presented in Appendix 1.

### Year of publication

**Fig. 2:**
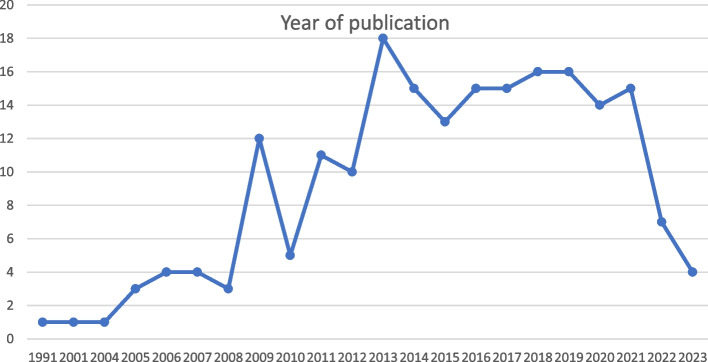
Number of included studies per year

Figure 2 shows the distribution of published studies from 1991 to 2023; with 2013, being the year with the highest number of publications, and 148 of the 203 included studies being published since this year.

### Study methods

In total, 87% (*n* = 176) of the included studies were intervention studies. Of these, 41 studies were randomized controlled trials. The remaining publications were explorative studies (*n* = 24) and observational studies (*n* = 3).

### Assessment tools

All included studies used assessment tools to evaluate NTS and TS.

**Fig. 3:**
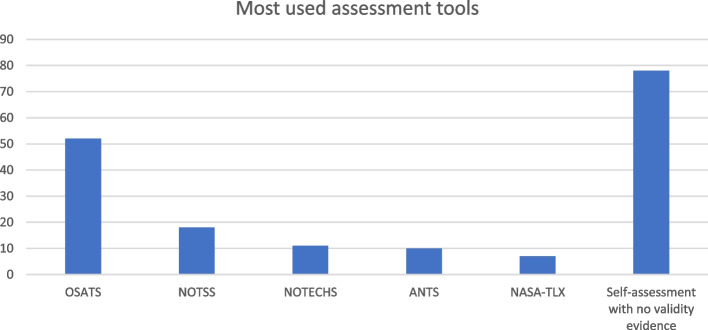
Frequently used assessment tools

As shown in Fig. 3, Objective Structured Assessment of Technical Skills (OSATS) was the most frequently used assessment tool for TS. OSATS measures tissue handling, time and motion, instrument handling, knowledge of instruments, overall flow during the procedure, and knowledge of the procedure [36]. Sixteen studies used variations of OSATS to assess TS [37], but only 9 of these used variations including validity evidence. For the remaining 7 studies, reporting on validity evidence was unspecified or non-validated. Additionally, nine studies used a procedure-specific checklist or assessment tool based on OSATS to assess TS, whereof 5 of 9 included validity evidence. (Table 1).

**Table 1:** OSATS and variations of OSATS

	Validity evidence	No validity evidence
**OSATS**	52	-
**Modified OSATS and variation thereof**	9	7
**Tool based on OSATS**	5	4

For assessment of NTS, the Non-technical Skills for Surgeons (NOTSS) tool [38] was the most frequently used assessment tool (*n* = 18). NOTSS measures situational awareness, decision making, communication & teamwork, and leadership in a surgical setting [38].

A total of 72 studies used tools with strong validity evidence to assess both TS and NTS. However, 62 studies used only one assessment tool with validity evidence in their study to assess either NTS or TS, while the assessment of the other was either non-validated or non-specified. Self-assessment in various forms was the most frequently used form of assessment (*n* = 78) which was often used to assess the participants’ NTS either in a survey or questionnaire.

### Medical specialties represented

**Fig. 4:**
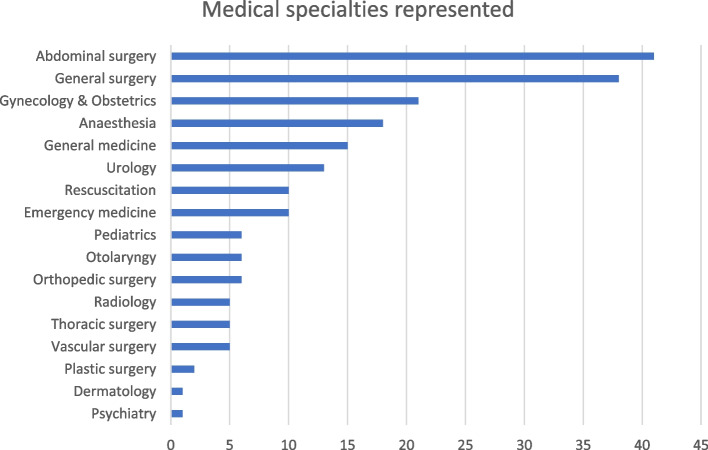
Number of studies within each medical specialty

Abdominal surgery was the most frequent medical specialty represented in the included publications (*n* = 47). However, most of the studies did not specify what kind of surgery or medical specialty they assessed. As such, they are represented in “general surgery” or “general medicine”. Resuscitation was categorized independently of medical specialty as it involves different specialties (Fig. 4).

### Correlation between NTS and TS

The objective of this review was to map to which degree the current literature investigates technical and non-technical skills. To this end, we found 203 articles that assessed both skills, but only 46 studies tested for a correlation. Here, the majority found a positive correlation, supporting our assumption that technical and non-technical skills correlate.

The 46 studies that tested for correlation between NTS and TS often used Spearman regression or Pearson *r* correlation. Forty of these (87%) found a positive correlation. The remaining six publications did not find any or found a negative correlation when testing the association between the two skill sets.

Studies that found a positive correlation investigated general surgical skills (*n* = 15), laparoscopic skills (*n* = 13), crisis and trauma skills (*n* = 9), diagnostic skills (*n* = 2), and robotic surgery (*n* = 1) within for example paediatrics, urology, OBGYN, and various surgical specialties. The methodology in the 40 studies that found a positive correlation were intervention studies (*n* = 33), explorative studies (*n* = 6), and one was an observational study. Within studies on general surgery (*n* = 9), results showed that NOTSS or NOTECHS had a positive correlation with surgical performance. In the resuscitation-based studies (*n* = 7), a positive correlation was found between leadership, teamwork, and situational awareness and technical resuscitation skills.

In general, the most common NTS tested for in the studies that found a positive correlation between NTS and TS, were leadership (*n* = 14) and decision making (*n* = 14). However, situational awareness (*n* = 12), teamwork (*n* = 12), and communication (*n* = 11) were also commonly tested for in the studies. The most frequently assessed TS with a correlation to NTS was surgical performance (*n* = 27), either in a simulation-based setting or in the OR.

## Discussion

While several studies aimed to assess both TS and NTS, most of the identified studies did not provide an assessment of both constructs, showcasing a lack of methodological rigour [32, 39]. This finding might reflect a broader issue: a limited understanding of how TS and NTS are integrated to influence clinical activities. Our findings support this argument, highlighting not only the absence of a clear, concise definition of NTS but also the insufficient statistical analysis in the current research. Studies by Cleland et al. and Tsuda et al. show how the acquisition of TS happens in relation to other learners and teachers [21, 40]; thereby highlighting that learning of TS is influenced by interpersonal and cognitive skills. In continuation of this, the distinction between the two skill sets might seem arbitrary. This is supported by socio-cultural educational studies emphasizing that the dispositions of the learner and the affordances of the environment form the outcome of learning [23, 41, 42]. These studies were, however, qualitative and cannot establish whether a clear relationship between TS and NTS exists. Within the quantitative studies we saw a lack of theoretical presentation or discussion of how TS and NTS interacts in clinical activities. This gap between the investigation and the theoretical point of departure may impact the development of assessment tools to guide such integration of TS and NTS, and thereby the investigation of the effect of these tools.

While studies conducted outside medical education may point to a correlation and interdependence between NTS and TS [16, 17], the majority of included studies in the present review did not investigate such an interdependence. Of the 192 included manuscripts, we identified 149 studies that did not test for correlation between NTS and TS despite including both skill sets in their analysis. However, many of these speculate if the two skill sets influence each other. The majority of these 149 studies included an intervention based on NTS, which resulted in better technical performance among the participants – but they did not statistically analyse this association and only interpreted its efficiency based on the improvement of TS. To establish how an improvement in TS is influenced by NTS, future studies should simultaneously assess both skill sets and thereby investigate if certain aspects of NTS influence TS and vice versa. This is especially relevant considering the surge of studies on self-regulated learning. In self-regulated learning studies, one of the most frequently used models, the circular model by Zimmerman, sees behaviour as a result of an interplay the individual learner and the environment. Similar is seen in the literature on motor learning arguing that motor control is a result of continues cognitive adaptation to the environment rather than a muscle memory distinct from cognition [43]. Many of these thoughts arise from the insights from Gibson, arguing that different sources of perceptual information present different affordances for learners to execute specific actions [44]. Here, our perception of the environment and task, influence how we execute motor behaviour. This is exemplified in an often-used quote from Gibson (1979, p. 223); *“we must perceive in order to move*,* but we must also move in order to perceive.”*. Similarly, Ziegler (2011) argues that learning of new technical skills happens in a way that take the learners intention, attention, and adjustment into account. Here Ziegler highlights self-regulated learning as a useful framework, as the focus on individual and environmental aspects within this framework is complementary and the learning of skills in self-regulated learning can be understood as adaptations to the respective environments [45–47].

Together these theoretical lenses might help future research in medical education, that aim to investigate how different elements of specific skill sets influence or correlate with each other.

Importantly, of the remaining 43 studies that investigated the correlation between TS and NTS, we found that the majority showed a positive correlation, further incentivizing future studies in medical education, and medical educators to account the interdependence of technical and non-technical skills. However, future research should also account for a reconceptualization of non-technical skills, as we also identified common issues with this category during our data retrieval and analysis.

### Is knowledge a skill?

During our analysis, we found several publications that defined knowledge as a NTS and mentioned knowledge as a part of their NTS training program. While knowledge is important in medical practice, it seems reasonable to argue that the underlying construct does not theoretically, nor empirically, apply as a skill [48–50]. In this sense, knowledge refers to the process of expanding a frame of mind, rather than an applicable skill [51, 52]. Thus, we argue that knowledge more so is the foundation we base our actions on, rather than being a skill itself. This points to the notion that NTS, in general, is poorly defined and therefore harder, than TS, to assess. We excluded studies that defined knowledge as NTS, without assessing any other aspects of NTS.

Despite a vast amount of research aiming to develop behavioural markers [2, 38, 53], seeking consensus on how to identify and define NTS [18], and systematically reviewing and developing taxonomies or frameworks for NTS [54–56], there is still little consensus on how to assess NTS [4, 57]. This issue affects the ability to compare results across studies, which is also evident in the results of this review as we found that only few publications used tools with validity evidence to assess NTS.

### Self-assessment

Our results showed that self-assessment, without any description of validity evidence, was often used to evaluate NTS. Subjective assessment is less reliant than validated psychometric tools or assessment tools used by external assessors [58]. Most common was self-reported confidence on a Likert scale in pre- and post-evaluations. However, psychometric tools assessing mental strain, anxiety, perceived level of competency, comfort, and self-efficacy were also frequently used.

While the accuracy of self-assessment seems to increase with experience [59], there is still a lack of description of validity evidence in tools for self-assessment of NTS amongst novices, where the impact of NTS are arguably most evident [60–63]. Furthermore, Ladonna et al. [64] found that self-doubt affects clinicians at all career stages and emphasize that medical culture must create space for physicians to share their struggles. This highlights the issue of relying on self-assessment as this tool is influenced by the learning culture that the learners are a part of. Hence, it is recommended that research validate the tools they use to assess NTS, using well established frameworks such as Kane’s validity framework [65, 66].

### Limitations

This study has several limitations. Firstly, when conducting a review, the identification of all relevant literature on the subject is of essence. To ensure this, advice on constructing a sound literature search was given by a librarian with extensive experience. Additionally, publications not initially identified through the literature search were later found through manual searching, suggesting that our search may not have captured all relevant studies. However, despite our efforts to exhaust all relevant literature, some relevant studies, like a study by Gjeraa et al. who specifically investigated this relationship [67], still evaded our investigation. Unclear definitions of NTS in the literature provided an obstacle to the investigation of the relationship between TS and NTS in the present study. This was evident in that some non-technical assessment tools (i.e., the LBDQ) were not identified in this search. To overcome this obstacle, we applied a broad definition of NTS defined as all skills that are not body-kinetic skills but excluded studies that defined knowledge as NTS without assessing other NTS. Hence, the present study incorporated personal resources skills and cognitive skills in addition to skills such as teamwork and communication which are traditionally defined as NTS.

## Conclusions

We aimed to explore the association between NTS and TS. We found that the two constructs showed to influence each other in the majority of the studies identified. This indicates that NTS and TS are interrelated constructs, which is in accordance with much literature on motor learning and cognitive sciences. However, we also found that there is a need for stricter conceptualisation of NTS, and that this construct is often not formally assessed in medical education. As our results suggest, there is a need for better conceptualization of non-technical skills, or rather, a delineation of what these human factors are.

## Supplementary Information

Supplementary Material 1.

Supplementary Material 2.

## Data Availability

The author confirms that all data generated or analysed during this study are included in this published review article.
