# Supplementary material for: Training technical or non-technical skills: an arbitrary distinction? A scoping review
Source: BMC Med Educ. 2024 Dec 18;24:1451. doi: 10.1186/s12909-024-06419-6 (PMC11654166; doi:10.1186/s12909-024-06419-6)
Supplement: Supplementary file 2 — Supplementary Material 2. [file 12909_2024_6419_MOESM2_ESM.docx]

**Appendix 1**

| **Author** | **Title** | **Year** | **Correlation between  TS and NTS** |
| --- | --- | --- | --- |
| Abdelshehid, C. S. et. al. | High-Fidelity Simulation-Based Team Training in Urology: Evaluation of Technical and Nontechnical Skills of Urology Residents During Laparoscopic Partial Nephrectomy | 2013 | - |
| Abe, T. et. al. | The effect of repeated full immersion simulation training in ureterorenoscopy on mental workload of novice operators | 2019 | - |
| Agarwal, A. et. al. | Improving knowledge, technical skills, and confidence among pediatric health care providers in the management of chronic tracheostomy using a simulation model | 2015 | - |
| Ahsin, S. et. al. | Student led outreach workshops to promote basic life support | 2021 | - |
| Ahlborg, L. et. al. | Simulator training and non-technical factors improve laparoscopic performance among OBGYN trainees | 2013 | + |
| Ahlborg, L. et. al. | Non-technical factors influence laparoscopic simulator performance among OBGYN residents | 2012 | + |
| Alabi, O. et. al | Introduction of an ENT emergency-safe boot camp into postgraduate surgical training in the Republic of Ireland | 2021 | - |
| Alici, F. et. al. | Objective Structured Assessment of Technical Skills (OSATS) evaluation of hysteroscopy training: a prospective study | 2014 | - |
| Anderson, J. M. et. al. | Simulating Extracorporeal Membrane Oxygenation Emergencies to Improve Human Performance. Part II: Assessment of Technical and Behavioral Skills | 2006 | - |
| Argun, O. B. et. al | Multi-Institutional Validation of an OSATS for the Assessment of Cystoscopic and Ureteroscopic Skills | 2015 | - |
| Arora, S. et. al. | Stress impairs psychomotor performance in novice laparoscopic surgeons | 2010 | + |
| Arora, S. et. al. | Mental Practice Enhances Surgical Technical Skills: A Randomized Controlled Study | 2011 | + |
| Arora, S. et. al | Self vs expert assessment of technical and non-technical skills in high fidelity simulation | 2011 | - |
| Bacarese-Hamilton, J. et. al. | Simulation in the Early Management of Gastroschisis | 2013 | - |
| Back, S. J. et al. | Ultrasound tutorials in under 10 minutes: Experience and results | 2016 | - |
| Balki, M. et al. | Effectiveness of using high-fidelity simulation to teach the management of general anesthesia for Cesarean delivery | 2014 | + |
| Barra, F. L. et al. | Anesthesiology Resident Induction Month: a pilot study showing an effective and safe way to train novice residents through simulation | 2018 | - |
| Barre, J. et al. | Midwifery students’ retention of learning after screen-based simulation training on neonatal resuscitation: a pilot study | 2020 | - |
| Bedetti, B. et al. | Virtual simulation and learning new skills in video-assisted thoracic surgery | 2018 | - |
| Biron, V. L. et al. | Teaching Cricothyrotomy: A Multisensory Surgical Education Approach for Final-Year Medical Students | 2013 | - |
| Black, S. A. et al. | Assessment of surgical competence at carotid endarterectomy under local anaesthesia in a simulated operating theatre | 2010 | + |
| Bohnen, J. D. et al. | High-Fidelity Emergency Department Thoracotomy Simulator With Beating-Heart Technology and OSATS Tool Improves Trainee Confidence and Distinguishes Level of Skill | 2018 | - |
| Boyle, E. et al. | Coping with stress in surgery: The difficulty of measuring non-technical skills | 2011 | * |
| Brunckhorst, O. et al. | The Relationship Between Technical And Nontechnical Skills Within A Simulation-Based Ureteroscopy Training Environment | 2015 | + |
| Brunckhorst, O. et al. | Simulation-based ureteroscopy skills training curriculum with integration of technical and non-technical skills: a randomised controlled trial | 2015 | - |
| Buerkle, B. et al. | Objective structured assessment of technical skills evaluation of theoretical compared with hands-on training of shoulder dystocia management: A randomized controlled trial | 2014 | - |
| Buerkle B. et al. | Objective Structured Assessment of Technical Skills (OSATS) evaluation of theoretical versus hands-on training of vaginal breech delivery management: A randomized trial | 2013 | - |
| Caskey, R. C. et al. | Integration of Hands-On Team Training into Existing Curriculum Improves Both Technical and Nontechnical Skills in Laparoscopic Cholecystectomy | 2017 | + |
| Chowriappa, A. et al. | Augmented-reality-based skills training for robot-assisted urethrovesical anastomosis: a multi-institutional randomised controlled trial | 2015 | - |
| Chudnoff, S. G. et al. | Efficacy of a novel educational curriculum using a simulation laboratory on resident performance of hysteroscopic sterilization | 2010 | - |
| Cohen, D. et al. | Tactical and operational response to major incidents: Feasibility and reliability of skills assessment using novel virtual environments | 2013 | - |
| Couper, K. et al. | Training approaches for the deployment of a mechanical chest compression device: A randomised controlled manikin study | 2018 | - |
| Criss, C. N. et al. | Evaluating a Solely Mechanical Articulating Laparoscopic Device: A Prospective Randomized Crossover Study | 2019 | - |
| Desender, L. et al. | A Multicentre Trial of Patient specific Rehearsal Prior to EVAR: Impact on Procedural Planning and Team Performance | 2017 | - |
| Domingues, R. C. L. et al. | Global overall rating for assessing clinical competence: What does it really show? | 2009 | - |
| Dreyer, J. et al. | Teaching the Management of Surgical Emergencies Through a Short Course to Surgical Residents in East/Central Africa Delivers Excellent Educational Outcomes | 2013 | - |
| Edwards, T. C. et al. | Immersive virtual reality enables technical skill acquisition for scrub nurses in complex revision total knee arthroplasty | 2021 | - |
| Edwards, T. C. et al. | Collaborative Team Training in Virtual Reality is Superior to Individual Learning For Performing Complex Open Surgery: A Randomized Controlled Trial | 2023 | + |
| Everett, T. C. et al. | The impact of critical event checklists on medical management and teamwork during simulated crises in a surgical daycare facility | 2016 | - |
| Fernandez, G. L. et al. | Boot Camp: Educational Outcomes After 4 Successive Years of Preparatory Simulation-Based Training at Onset of Internship | 2012 | - |
| Gasteratos, K. et al. | Video-Assisted Simulation Training in Burn Management: A Comparative Cohort Study on the Assessment of Technical and Non-technical Competencies | 2021 | - |
| Gannon, S. J. et al. | Do resident's leadership skills relate to ratings of technical skill? | 2016 | + |
| Gawad, N. et al. | Introduction of a comprehensive training curriculum in laparoscopic surgery for medical students: A randomized trial | 2014 | - |
| Geeraerts, T. et al. | Physiological and self-assessed psychological stress induced by a high fidelity simulation course among third year anesthesia and critical care residents: An observational study | 2017 | - |
| Goldenberg, M. G. et al. | Simulation-Based Laparoscopic Surgery Crisis Resource Management Training—Predicting Technical and Nontechnical Skills | 2018 | + |
| Govender, K. et al. | Comparison of two training programmes on paramedic-delivered CPR performance | 2016 | - |
| Gulati, G. et al. | Musculoskeletal ultrasound in internal medicine residency – a feasibility study | 2015 | - |
| Han, B.J. et al. | The use of advanced robotic simulation labs to advance and assess senior resident robotic skills and operating room leadership competency: a pilot study | 2022 | - |
| Hedman, L. et al | Visual working memory influences the performance in virtual image–guided surgical intervention | 2007 | + |
| Hendrickx, K. et al. | Learning intimate examinations with simulated patients: The evaluation of medical students' performance | 2009 | - |
| Heskin, L. et al. | The impact of a surgical boot camp on early acquisition of technical and nontechnical skills by novice surgical trainees | 2015 | - |
| Hicks Jr, G. L. et al. | Cardiopulmonary bypass simulation at the Boot Camp | 2011 | - |
| Hilal, Z. et al. | A randomized comparison of video demonstration versus hands-on training of medical students for vacuum delivery using Objective Structured Assessment of Technical Skills (OSATS) | 2017 | - |
| Hu, Y. et al. | Video Self-Assessment of Basic Suturing and  Knot Tying Skills by Novice Trainees | 2013 | * |
| Innocenti, F. et al. | Improving technical and non-technical skills of emergency medicine residents through a program based on high-fidelity simulation | 2022 | - |
| Izawa, Y. et al. | Ex-vivo and live animal models are equally effective training for the management of a penetrating cardiac injury | 2016 | - |
| Jabbour, N. et al. | Psychomotor skills training in pediatric airway endoscopy simulation | 2011 | - |
| Johnston, M. J. et al. | Improving Escalation of Care: A Double-blinded Randomized Controlled Trial | 2016 | - |
| Karmali, R. J. et al. | The Surgical Skills and Technology Elective Program (SSTEP): A comprehensive simulation-based surgical skills initiative for preclerkship medical students | 2018 | - |
| Kelly, M. A. et al. | Empowering the registered nurses of tomorrow: Students' perspectives of a simulation experience for recognising and managing a deteriorating patient | 2014 | - |
| Kenny, L. et al. | Training cardiothoracic surgeons of the future: The UK experience | 2018 | - |
| Kohls-Gatzoulis, J. A. et al. | Teaching cognitive skills improves learning in surgical skills courses: A blinded, prospective, randomized study | 2004 | * |
| Krage, R. et al | Relationship between non-technical skills and technical performance during cardiopulmonary resuscitation: does stress have an influence? | 2017 | + |
| Kumar, A. et al. | Evaluation of learning from Practical Obstetric Multi-Professional Training and its impact on patient outcomes in Australia using Kirkpatrick's framework: a mixed methods study | 2018 | - |
| Kurien, G. et al. | Can a multisensory teaching approach impart the necessary knowledge, skills, and confidence in final year medical students to manage epistaxis? | 2013 | - |
| Lambden, S. et al. | The Imperial Paediatric Emergency Training Toolkit (IPETT) for use in paediatric emergency training: Development and evaluation of feasibility and validity | 2013 | + |
| LeBlanc, V. et al. | Examination stress leads to improvements on fundamental technical skills for surgery | 2008 | - |
| LeBlanc, V. R. et al. | Psychometric properties of an integrated assessment of technical and communication skills | 2009 | * |
| Lee, Jason Y. et al. | High Fidelity Simulation Based Team Training in Urology: A Preliminary Interdisciplinary Study of Technical and Nontechnical Skills in Laparoscopic Complications Management | 2012 | - |
| Lenchus, J. D. et al. | Filling the Void: Defining Invasive Bedside Procedural Competency for Internal Medicine Residents | 2013 | - |
| Levinson, M. et al. | Description and student self-evaluation of a pilot integrated small group learning and simulation programme for medical students in the first clinical year | 2016 | - |
| Liao, C. H. et al. | Video Coaching Improving Contemporary Technical and Nontechnical Ability in Laparoscopic Education | 2020 | - |
| Liu, K. J. et al. | A video-based, flipped classroom, simulation curriculum for dermatologic surgery: A prospective, multi-institution study | 2019 | - |
| Louridas, M. et al. | Randomized clinical trial to evaluate mental practice in enhancing advanced laparoscopic surgical performance | 2014 | + |
| MacArthur, S. et al. | Effect of a Spay Simulator on Student Competence and Anxiety | 2021 | - |
| Malekzadeh, S. et al. | A Model for Training and Evaluation of Myringotomy and Tube Placement Skills | 2011 | - |
| Manley, K. et al. | Hybrid simulation compared to manikin alone in teaching pelvic examinations: a randomised control trial | 2016 | - |
| Mannella, P. et al. | Simulation of childbirth improves clinical management capacity and self-confidence in medical students | 2018 | - |
| Mannella, P. et al. | Effect of high-fidelity shoulder dystocia simulation on emergency obstetric skills and crew resource management skills among residents | 2016 | - |
| Maslekar, S. et al | Patient satisfaction with lower gastrointestinal endoscopy: doctors, nurse and nonmedical endoscopists | 2009 | - |
| Maschuw, K. et al. | Do Soft Skills Predict Surgical Performance? | 2011 | + |
| McAnena, P. F. et al. | Undergraduate basic surgical skills education: impact on attitudes to a career in surgery and surgical skills acquisition | 2017 | - |
| McCulloch, P. et al. | The effects of aviation-style non-technical skills training on technical performance and outcome in the operating theatre | 2009 | + |
| McCulloch, P. et al. | Combining systems and teamwork approaches to enhance the effectiveness of safety improvement interventions in surgery: The safer delivery of surgical services (S3) program | 2017 | - |
| McVey, R. M. et al. | Introduction of a Structured Assessment of Clinical Competency for Fellows in Gynecologic Oncology: A Pilot Study | 2015 | - |
| Mishra, A. et al. | The influence of non-technical performance on technical outcome in laparoscopic cholecystectomy | 2008 | + |
| Mishra, A. et al. | The Oxford NOTECHS system: reliability and validity of a tool for measuring teamwork behaviour In the operating theatre | 2009 | + |
| Michelet, D. et al. | Effect of Computer Debriefing on Acquisition and Retention of Learning After Screen-Based Simulation of Neonatal Resuscitation: Randomized Controlled Trial | 2020 | - |
| Minneti, M. et al. | The Development of a Novel Perfused Cadaver Model With Dynamic Vital Sign Regulation and Real-World Scenarios to Teach Surgical Skills and Error Management | 2018 | - |
| Mitric, C. et al. | Impact of a Multidimensional Technical Skills Training Session Before Obstetrics and Gynaecology Clerkship Rotation on Performance and Exposure | 2018 | - |
| Miyasaka, K. W. et al. | Development and Implementation of a Clinical Pathway Approach to Simulation-Based Training for Foregut Surgery | 2015 | - |
| Moorthy, K. et al. | A Human Factors Analysis of Technical and Team Skills Among Surgical Trainees During Procedural Simulations in a Simulated Operating Theatre | 2005 | + |
| Moorthy, K. et al. | Self-assessment of performance among surgical trainees during simulated procedures in a simulated operating theater | 2006 | - |
| Moorthy, K. et al. | Surgical Crisis Management Skills Training and Assessment | 2006 | - |
| Morgan, L. et al. | A combined teamwork training and work standardisation intervention in operating theatres: Controlled interrupted time series study | 2014 | - |
| Moulton, C-A. et al. | Teaching communication skills using the integrated procedural performance instrument (IPPI): a randomized controlled trial | 2009 | - |
| Mudumbai, S. C. et al. | External Validation of Simulation-Based Assessments With Other Performance Measures of Third-Year Anesthesiology Residents | 2012 | - |
| Müller, M. P. et al. | Excellence in performance and stress reduction during two different full scale simulator training courses: A pilot study | 2009 | - |
| Munabi, N. C. O. et al. | Pilot Evaluation of the Impact of a Mission-Based Surgical Training Rotation on the Plastic Surgery Skills and Competencies Development of General Surgery Residents in Rwanda | 2019 | - |
| Nakazato, T. et al. | A 1-day simulation-based boot camp for incoming general surgery residents improves confidence and technical skills | 2019 | - |
| Nathan, A. et al. | Virtual Interactive Surgical Skills Classroom: A Parallel-group, Non-inferiority, Adjudicator-blinded, Randomised Controlled Trial (VIRTUAL) | 2022 | - |
| Nathwani, J. et al. | Relationship Between Technical Errors and Decision-Making Skills in the Junior Resident | 2016 | + |
| Neal, J. M. et al. | ASRA Checklist Improves Trainee Performance During a Simulated Episode of Local Anesthetic Systemic Toxicity | 2012 | - |
| Nelson, B. et al. | Playing the Surgical Technologist Role by Surgery Residents Improves Their Technical and Nontechnical Skills | 2019 | - |
| Nguyen, L. H. P. et al. | Managing the airway catastrophe: longitudinal simulation-based curriculum to teach airway management | 2019 | - |
| Nihira, M. A. et al. | Training community gynecologists to perform intraoperative cystoscopy: A competency-based training experience | 2014 | - |
| Nikendei, C. et al. | Integration of role-playing into technical skills training: A randomized controlled trial | 2007 | - |
| Nishisaki, A. et al. | A multi-institutional high-fidelity simulation "boot camp" orientation and training program for first year pediatric critical care fellows | 2009 | - |
| Norris, S. et al. | Effect of a Surgical Teaching Video on Resident Performance of a Laparoscopic Salpingo-oophorectomy: A Randomized Controlled Trial | 2020 | - |
| Noveanu, J. et al. | Assessment of simulated emergency scenarios: Are trained observers necessary? | 2017 | - |
| O'Keeffe, D. A. et al. | Use of a Novel Measure of Nontechnical Skills in Surgical Trainees: Is There an Association With Technical Skills Performance? | 2019 | + |
| Olson, T. P. et al. | A simulation-based curriculum can be used to teach open intestinal anastomosis | 2012 | - |
| Oshiro, K. et al. | A structured program for teaching pancreatojejunostomy to surgical residents and fellows outside the operating room: a pilot study | 2021 | - |
| Pade, K. H. et al. | The Efficacy of a Brief Educational Training Session in Point-of-Care Pediatric Hip Ultrasound | 2022 | * |
| Paige, J. T. et al. | Thinking it Through: Mental Rehearsal and Performance on 2 Types of Laparoscopic Cholecystectomy Simulators | 2015 | + |
| Palter, V. N. et al. | Validation of a structured training and assessment curriculum for technical skill acquisition in minimally invasive surgery: A randomized controlled trial | 2013 | - |
| Partamin et al. | Patterns in training, knowledge, and performance of skilled birth attendants providing emergency obstetric and newborn care in Afghanistan | 2012 | - |
| Patrawalla, P. et al. | A Regional, Cost-Effective, Collaborative Model for Critical Care Fellows’ Ultrasonography Education | 2019 | - |
| Peltonen, V. et al. | Randomized controlled trial comparing pit crew resuscitation model against standard advanced life support | 2022 | - |
| Pender, C. et al. | All for knots: evaluating the effectiveness of a proficiency-driven,  simulation-based knot tying and suturing curriculum for medical students during their third-year surgery clerkship | 2017 | - |
| Perez-Daniel, I. J. et al. | Open surgery performance evaluation in undergraduate medicine  students with a projection to undergo a surgical specialty training | 2020 | - |
| Pfandler, M. et al. | Technical and Nontechnical Skills in Surgery: A Simulated Operating Room Environment Study | 2019 | + |
| Phitayakorn, R et al. | The relationship between intraoperative teamwork and management skills in patient care | 2015 | * |
| Pierre, R. B. et al. | Student self-assessment in a paediatric objective structured clinical examination | 2005 | - |
| Pikoulis, E. et al. | When the Going Gets Tough, the Tough Get Going: Improving the  Disaster Preparedness of Health Care Providers: A Single Center’s 4-Year Experience | 2020 | - |
| Piquette, D. et al. | Resident competencies before and after short intensive care unit rotations: a multicentre pilot observational study | 2020 | - |
| Pittelkow, T. P. et al. | Pain medicine fellow neuromodulation surgical skill assessment tool: a pilot | 2019 | - |
| Ponton-Carss, A. et al. | Assessment of technical and nontechnical skills in surgical residents | 2016 | + |
| Posner, G. D. and  Hamstra, S. J. | Too much small talk? Medical students' pelvic examination skills falter with pleasant patients | 2013 | + |
| Powers, K. A. et al. | Simulated laparoscopic operating room crisis: An approach to enhance the surgical team performance | 2008 | - |
| Prakash, S. et al. | Prospective Randomized Controlled Trial of Video- Versus Recall-Assisted Reflection in Simulation-Based Teaching on Acquisition and Retention of Airway Skills among Trainees Intubating Critically Ill Patients | 2020 | - |
| Pucher, P. H. et al. | Randomized clinical trial of the impact of surgical ward-care checklists on postoperative care in a simulated environment | 2014 | - |
| Pucher, P. H. et al. | Ward simulation to improve surgical ward round performance: A randomized controlled trial of a simulation-based curriculum | 2014 | - |
| Pucher, P. H. et al. | Virtual-world hospital simulation for real-world disaster response:  Design and validation of a virtual reality simulator for mass casualty incident management | 2014 | - |
| Pugh, C. et al. | Outcome measures for surgical simulators: Is the focus on technical skills the best approach? | 2010 | + |
| Pugh, D. et al. | A procedural skills OSCE: assessing technical and non-technical skills of internal medicine residents | 2015 | + |
| Rabheru, K. et al. | Comparison of traditional didactic seminar to high-fidelity simulation for teaching electroconvulsive therapy technique to psychiatry trainees | 2013 | - |
| Raffaeli, G. et al. | Start a Neonatal Extracorporeal Membrane Oxygenation Program: A Multistep Team Training | 2018 | - |
| Raison, N. et al. | Cognitive training for technical and non-technical skills in robotic surgery: a randomised controlled trial | 2018 | - |
| Rajesh, A. et al. | International Medical Graduates are Comparable to American Medical Graduates as General Surgery Interns | 2021 | - |
| Ramjeeawon, A. et al. | Using Fully-Immersive Simulation Training with Structured Debrief to Improve Nontechnical Skills in Emergency Endovascular Surgery | 2020 | - |
| Rao, R. et al. | Can Simulated Team Tasks be Used to Improve Nontechnical Skills in the Operating Room? | 2016 | + |
| Ray, Juliet J. et al. | Association Between American Board of Surgery In-Training Examination Scores and Resident Performance | 2016 | - |
| Reisner, E. et al. | A model for the assessment of students' physician-patient interaction skills on the surgical clerkship | 1991 | - |
| Roberts, S.I. et al. | The Relationship Between Technical Skills, Cognitive Workload, and Errors During Robotic Surgical Exercises | 2022 | + |
| Rosen, L. et al. | Resident simulation training improves operative time of the retropubic midurethral sling procedure for stress incontinence | 2019 | - |
| Rovamo, L. et al. | Assessment of newborn resuscitation skills of physicians with a simulator manikin | 2011 | + |
| Rubio-Gurung, S. et al. | In situ simulation training for neonatal resuscitation: An RCT | 2014 | - |
| Sahovaler, A. et al. | Novel minimally invasive transoral surgery bleeding model implemented in a nationwide otolaryngology emergencies bootcamp | 2019 | - |
| Sakamoto, Y. et al. | Hands-on Simulation versus Traditional Video-learning in Teaching Microsurgery Technique | 2017 | + |
| Saraf, S. et al. | The relationship of praise/criticism to learning during obstetrical simulation: a randomized clinical trial | 2014 | - |
| Satterwhite, T. et al. | The stanford microsurgery and resident training (SMaRT) scale: Validation of an on-line global rating scale for technical assessment | 2014 | + |
| Savage, C. et al. | Safer paediatric surgical teams: A 5-year evaluation of crew resource management implementation and outcomes | 2017 | - |
| Sawyer, T. et al. | Correlations between technical skills and behavioral skills in simulated neonatal resuscitations | 2014 | + |
| Schijven, M. P. et al. | Transatlantic comparison of the competence of surgeons at the start of their professional career | 2010 | - |
| Schmidt, M. W. et al. | Self-directed training with e-learning using the first-person perspective for laparoscopic suturing and knot tying: a randomised controlled trial | 2019 | + |
| Schoeff, S. et al. | Microvascular anastomosis simulation using a chicken thigh model: Interval versus massed training | 2017 | - |
| Shah, A. et al. | Simulation to develop tomorrow’s medical registrar | 2013 | + |
| Shah, M. et al. | The role of cognitive training in endourology: A randomized controlled trial | 2018 | - |
| Shen, Z. et al. | A Novel Clinical-Simulated Suture Education for Basic Surgical Skill:  Suture on the Biological Tissue Fixed on Standardized Patient Evaluated with Objective Structured Assessment of Technical Skill (OSATS) Tools | 2018 | - |
| Shore, E. M. et al. | Validating a standardized laparoscopy curriculum for gynecology residents: a randomized controlled trial | 2016 | - |
| Siegel, N. A. et al. | In situ medical simulation investigation of emergency department procedural sedation with randomized trial of experimental bedside clinical process guidance intervention | 2015 | - |
| Skertich, N. J. et al. | Pediatric Surgery Simulation-Based Training for General Surgery  Residents: Placement of a Silastic Silo for Gastroschisis | 2020 | - |
| Sree Kumar, E. J. et al. | Impact of repeated simulation on learning curve characteristics of residents exposed to rare life threatening situations | 2020 | - |
| Sridhar, S. et al. | Implementation of a Pediatric Early Warning Score to Improve Communication and Nursing Empowerment in a Rural District Hospital in Rwanda | 2020 | - |
| Srinivasan, M. et al. | Connoisseurs of care? Unannounced standardized patients' ratings of physicians | 2006 | - |
| Stefanidis, D. et al. | Developing a coaching mechanism for practicing surgeons | 2016 | - |
| Steinemann, S. et al. | Assessing teamwork in the trauma bay: Introduction of a modified "nOTECHS" scale for trauma | 2012 | + |
| Stolz, L. et al. | Multimodular Ultrasound Orientation: Residents’ Confidence and Skill in Performing Point-of-care Ultrasound | 2018 | - |
| Stroud, L. and Cavalcanti, R. B. | Hybrid Simulation for Knee Arthrocentesis: Improving Fidelity in Procedures Training | 2013 | - |
| Suh, I. H. et al. | Retention of fundamental surgical skills learned in robot-assisted surgery | 2011 | - |
| Sullivan, M. E. et al. | The use of cognitive task analysis to improve the learning of percutaneous tracheostomy placement | 2007 | - |
| Suzuki M. et al. | Mental workload during endoscopic sinus surgery is associated with surgeons' skill levels | 2023 | + |
| Swords, C. et al. | Multidisciplinary Tracheostomy Quality Improvement in the COVID-19 Pandemic: Building a Global Learning Community | 2021 | - |
| Szasz, P. et al. | Setting Performance Standards for Technical and Nontechnical Competence in General Surgery | 2017 | - |
| Takashima, Y. et al. | Use of pelvic model-based simulation for sacrospinous ligament fixation education in novice learners: a single-blinded randomized controlled trial | 2021 | - |
| Thorne, C. J. et al. | Feedback in advanced life support: A quality improvement initiative | 2020 | - |
| Undre, S. et al. | Multidisciplinary Crisis Simulations: The Way Forward for Training Surgical Teams | 2007 | - |
| van den Bos-Boon, A. et al. | Professional Assessment Tool for Team Improvement: An assessment tool for paediatric intensive care unit nurses' technical and nontechnical skills | 2022 | - |
| Van Empel, P. J. et al. | Open knot-tying skills: Resident skills assessed | 2013 | - |
| Von Wyl, T. et al. | Technical and non-technical skills can be reliably assessed during paramedic simulation training | 2009 | + |
| Weiskittel, T. M. et al. | Team-Based Ultrasound Objective Structured Practice Examination (OSPE) in the Anatomy Course | 2021 | - |
| Weiss, P. M. | How do medical student self-assessments compare with their final clerkship grades? | 2005 | - |
| Wetzel, C. M. et al. | Stress Management Training for Surgeons-A Randomized, Controlled, Intervention Study | 2011 | - |
| Whittle, S. R. and  Eaton, D. G. M. | Attitudes towards transferable skills in medical undergraduates | 2001 | - |
| Wiebracht, N. D. et al. | Pilot testing of a novel surgical simulator for endoscopic zenker's diverticulotomy | 2017 | - |
| Wild, C. et al. | Telestration with augmented reality for visual presentation of intraoperative target structures in minimally invasive surgery: a randomized controlled study | 2022 | - |
| Willaert, W. et al. | Efficient implementation of patient-specific simulated rehearsal for the carotid artery stenting procedure: Part-task rehearsal | 2011 | - |
| Williams, J. B. et al. | Intermethod reliability of real-time versus delayed videotaped evaluation of a high-fidelity medical simulation septic shock scenario | 2009 | - |
| Willuth, E. et al. | Robotic-assisted cholecystectomy is superior to laparoscopic cholecystectomy in the initial training for surgical novices in an ex vivo porcine model: a randomized crossover study | 2022 | - |
| Winkel, A. F. et al. | A Simple Framework for Assessing Technical Skills in a Resident Observed Structured Clinical Examination (OSCE): Vaginal Laceration Repair | 2013 | - |
| Wojcik, B. M. et al. | The Resident-Run Minor Surgery Clinic: A Four-Year Analysis of Patient Outcomes, Satisfaction, and Resident Education | 2021 | - |
| Wong, H. J. et al. | Teaching peroral endoscopic pyloromyotomy (POP) to practicing endoscopists: An “into-the-fire” approach to simulation | 2021 | - |
| Woo, M. et al. | Effectiveness of a novel training program for emergency medicine residents in ultrasound guided insertion of central venous catheters | 2009 | - |
| Wood, T.C. | Non-technical skills simulation-based training model for managing intraoperative posterior capsule rupture | 2023 | - |
| Wright, A. et al. | Supporting international medical graduates in rural Australia: a mixed methods evaluation | 2012 | - |
| Yee, A. et al. | The Effect of Surgical Video on Resident Performance of Carpal Tunnel Release: A Cadaveric Simulation-Based, Prospective, Randomized, Blinded Pilot Study | 2020 | - |
| Yeung, J. H. Y. et al. | Factors affecting team leadership skills and their relationship with quality of cardiopulmonary resuscitation | 2012 | + |
| Yoo, M.S. et al. | Simulation of Inpatient Medical Critical Events for Physicians at a Community Hospital | 2022 | - |
| Young, M. et al. | The Success and Evolution of a Urological “Boot Camp” for Newly Appointed UK Urology Registrars: Incorporating Simulation, Nontechnical Skills and Assessment | 2019 | - |
| Yule, S. et al. | Crew Autonomy During Simulated Medical Event Management on Long Duration Space Exploration Missions | 2023 | - |
| Zevin, B. et al. | Comprehensive simulation-enhanced training curriculum for an advanced minimally invasive procedure: a randomized controlled trial | 2017 | - |
| Zevin, B. et al. | Implementation and evaluation of a comprehensive proficiency-based curriculum in an advanced, minimally invasive procedure: a multi-institutional Canadian experience | 2019 | - |

* = a test was conducted but no correlation or a negative correlation was found.

+ = a test was conducted, and a positive correlation was found

- = no test was conducted
